# Boron-Doped NiCoCuMoMn High-Entropy Alloys for Enhanced Electrocatalytic Water Splitting: An Experimental and Computational Study

**DOI:** 10.1021/acsaem.5c02722

**Published:** 2025-12-03

**Authors:** Hossein Mahdavi, Maryam Mansoor, Onur Ergen, Uğur Ünal, Hadi Jahangiri

**Affiliations:** † Materials Science and Engineering, 52979 Koç University, Sariyer, Istanbul 34450, Turkiye; ‡ Energy Institute, 52971 Istanbul Technical University, Maslak, Istanbul 34469, Turkiye; § Department of Electronics and Communications Engineering, Istanbul Technical University, Maslak, Istanbul 34469, Turkiye; ∥ Koç University Surface Science and Technology Center (KUYTAM), Koç University, Sariyer, Istanbul 34450, Turkiye; ⊥ Department of Chemistry, Koç University, Sariyer, Istanbul 34450, Turkiye; # Koç University Hydrogen Technologies Center (KUHyTech), Koç University, Sariyer, Istanbul 34450, Turkiye

**Keywords:** high-entropy alloys, boron doping, ball-milling, lattice strain engineering, electrocatalysis

## Abstract

High-entropy alloys offer a versatile platform for electrocatalysis, yet their optimization has so far been dominated by transition-metal compositional tuning. Here, we present the first demonstration of boron doping as a powerful nonmetal strategy to engineer high-entropy alloys for water splitting. Incorporating boron into NiCoCuMoMn HEAs drives a dramatic increase in the BCC phase fraction, refines crystallite sizes from the nanometer to subnanometer scale, and induces lattice distortions that create quasi-vacancy active sites. These unique structural modulations, validated by X-ray diffraction, Raman spectroscopy, and electron microscopy, are corroborated by first-principles calculations, showing that substitutional boron lowers oxygen adsorption energies and accelerates oxygen evolution reaction kinetics. As a result, the boron-doped HEA exhibits a breakthrough reduction in the oxygen evolution reaction overpotential (from 300 to 200 mV at 10 mA cm^–2^) and a sharp decrease in the Tafel slope (from 185 to 110 mV dec^–1^) while maintaining long-term stability over 48 h. Although the hydrogen evolution activity is moderately suppressed, this trade-off further confirms the boron-induced modulation of surface energetics. This combined experimental and theoretical study establishes boron doping as a design strategy for high-entropy alloy electrocatalysts, providing mechanistic evidence that nonmetal incorporation can rival metal compositional tuning in dictating catalytic performance.

## Graphical abstract

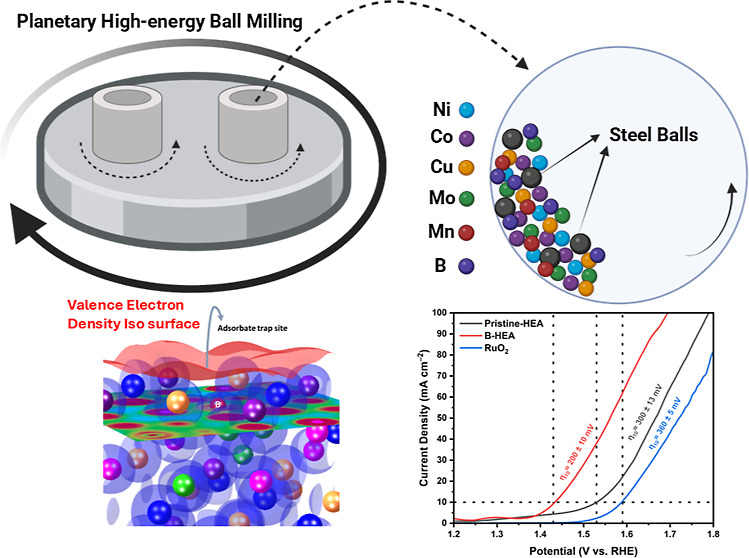

## 1 Introduction

High-entropy alloys (HEAs) are a novel class of materials composed of five or more principal elements, where high configurational entropy promotes the formation of highly stable solid solutions.^1–4^ Unlike traditional alloys, HEAs exhibit four core effects—high entropy, sluggish diffusion, severe lattice distortion, and the cocktail effect—which collectively enhance their stability and functional versatility.^5,6^ These effects impart unique material properties including exceptional mechanical,^7^ thermal,^1^ and catalytic properties^8^ surpassing those of conventional alloys.

These intrinsic properties have positioned HEAs as promising candidates for electrocatalysis, particularly in water splitting, an electrochemical process that decomposes water into hydrogen and oxygen using an external energy source.^1,6,9^ Hydrogen, as a clean and renewable fuel, holds great potential in sustainable energy applications, but its large-scale production depends on the development of efficient, cost-effective, and stable electrocatalysts. Noble metal-based catalysts such as Pt for the hydrogen evolution reaction (HER) and IrO_2_/RuO_2_ for the oxygen evolution reaction (OER) exhibit excellent activity but are limited by high cost, scarcity, and long-term stability issues.^1^ Extensive research efforts have been directed toward designing affordable and stable electrocatalysts to replace noble metal-based materials. Transition metal-based systems have attracted significant attention because of their remarkable catalytic activity, low production cost, and strong stability under alkaline conditions. As an example, Hojjati-Najafabadi et al. prepared a nickel–iron hydroxylphosphate electrocatalyst through a two-step electrodeposition approach, achieving low overpotentials of 161 mV for the HER and 273 mV for the OER at a current density of 10 mA cm^–2^.^10^

Because of their tunable phase composition, electronic structure, inherent stability, and multiactive sites, HEAs have emerged as a compelling alternative to conventional electrocatalysts.^11–13^ The electrocatalytic efficiency of HEAs is driven by their compositional complexity, which creates unique interactions between adjacent atoms and a continuous distribution of binding energies, leading to diverse adsorption sites and improved reaction kinetics.^11,14^ Additionally, sluggish diffusion decreases the reaction kinetics and the rate of phase transformation by preventing atomic migration, resistance to grain coarsening, which enhances the stability of electrocatalysts in a wide range of pH.^1,5^ Severe lattice distortion, arising from atomic size differences, introduces local strain fields that significantly influence mechanical, electronic, and superior catalytic properties.^6^ Furthermore, the cocktail effect enhances these properties by facilitating synergistic interactions between elements, crystal shape, phase boundaries, and phase distribution and tuning the electronic structure for optimized catalytic activity. These synergistic effects enabled HEAs to demonstrate remarkable electrocatalytic activity in water splitting reactions. For example, FeCoNiAlTi has been identified as an effective electrocatalyst for the HER due to its electronic synergy, while CrMnFeCoNi HEA exhibits superior OER performance due to lattice distortions and defect-driven reactivity.^15,16^ Additionally, Cu-containing HEAs, such as FeNiMnCrCu, have shown reduced activation energy for the OER due to electronic contributions from fully filled d orbitals of Cu, which allow electrons to transit to the d-band of other transition metals, reducing the d-vacancy number and thereby lowering the overall binding strength.^17^ He et al. fabricated nanoporous FeCoNiCuTi-based HEA electrocatalysts through chemical dealloying. The resulting porous structures with abundant active sites exhibited excellent bifunctional activity for both the HER and OER, attributed to the synergistic effect of multiple metals and the enhanced surface area.^18,19^

While HEAs inherently exhibit exceptional electrocatalytic properties, further optimization strategies are necessary to fine-tune their activity, stability, and reaction kinetics. Doping with nonmetal elements such as carbon,^20^ nitrogen,^21^ and boron^22^ has emerged as an effective approach to modulate electronic properties, enhance active site availability, and introduce beneficial lattice distortions. Among these, boron doping has proven to be an effective strategy for enhancing the OER by simultaneously tuning the electronic structure, defect chemistry, and surface reactivity of metal-based catalysts. Due to its unique electronegativity, boron can readily withdraw electrons from adjacent metal atoms, thereby inducing charge redistribution and promoting the adsorption of OH* intermediates.^23^ This electron-deficient environment facilitates the formation of oxygen vacancies^24^ and accelerates the active-phase reconstruction of metal hydroxides into high-valent oxyhydroxides such as β-NiOOH,^25,26^ which are widely recognized as the true active species during the OER. Consequently, boron doping offers a powerful and versatile route to boost catalytic kinetics, durability, and efficiency in transition-metal-based and HEA-based OER catalysts.^22^

Despite the promising catalytic properties of high-entropy borides,^27–29^ the specific effects of boron doping in HEAs remain largely unexplored. This study aims to systematically investigate the impact of boron incorporation in NiCoCuMoMn HEAs, focusing on its influence on phase distribution, microstructural evolution, and electrocatalytic performance in the OER and HER. To further support these findings, first-principles calculations based on density functional theory (DFT) are employed to assess the thermodynamic and electronic properties of boron-doped HEAs. The insights gained from this study will contribute to the rational design of advanced HEA-based catalysts for sustainable hydrogen production.

## 2 Materials and Methods

### 2.1 Materials

Nickel (Ni, Nanografi, 99.9%), cobalt (Co, Nanografi, 99.9%), copper (Cu, Nanografi, 99.9%), molybdenum (Mo, Nanografi, 99.9%), and manganese (Mn, Nanografi, 99.9%) micron powders, as well as amorphous nanoboron (B, Pavtec, 99.9%), were purchased and used without any further purification.

### 2.2 Sample Preparation

Homogeneous NiCoCuMoMn and B-doped NiCoCuMoMn HEA powders were synthesized through mechanical alloying by using elemental metal powders with a purity of 99.9%. Equiatomic proportions of Ni, Co, Cu, Mo, and Mn powders were mixed with introduced boron. The batches were prepared in two separate vials: one with the addition of amorphous nanoboron as a dopant at a weight fraction of 1.6 wt % and one without the addition. In the first step, powders were mixed for 30 min without adding the balls, and then the powders were mechanically alloyed for 20 h at 400 rpm, employing a ball-to-powder weight ratio (BPR) of 10:1 in a high-energy planetary ball mill (Retsch PM 200). The initial 10 h of milling was performed under a dry atmosphere, with the vials loaded and sealed in an argon-filled glovebox. The remaining 10 h was conducted in a wet atmosphere using ethanol as the milling medium. The powders were collected and dried at room temperature overnight. The NiCoCuMoMn HEA and B-doped NiCoCuMoMn are referred to as Pristine-HEA and B-HEA, respectively, throughout the rest of this text.

### 2.3 Structural and Compositional Characterization

The elemental composition of the synthesized powders was examined by using X-ray fluorescence (XRF) spectroscopy (Bruker S8 TIGER), which confirmed the presence of all intended elements with only minor deviations from the nominal values, as detailed in Table S1. However, boron could not be detected due to XRF’s limited sensitivity to low atomic number elements, and thus, its concentration was excluded from the analysis. Accordingly, inductively coupled plasma mass spectrometry (ICP–MS) was employed to quantify the boron in the system. Crystal structure characterization was performed using a Bruker D2 Phaser X-ray diffractometer (XRD) with Cu Kα radiation (λ = 1.5405 Å) across a 2θ range of 10–90°. Rietveld refinement was conducted using TOPAS Academics V5, applying a Gaussian function to model microstrain broadening and a Lorentzian function for particle size effects. Surface morphology and microstructural features were assessed via field emission scanning electron microscopy (FE-SEM, Zeiss Ultra Plus). High-resolution transmission electron microscopy (HRTEM, Hitachi HF5000) with aberration correction and integrated energy-dispersive X-ray spectroscopy (EDS) was used to further examine the crystallinity and elemental distribution at 200 kV. Surface chemical states were analyzed by X-ray photoelectron spectroscopy (XPS) using a Thermo Scientific K-α instrument with an Al Kα monochromatic source (1486.6 eV), and all spectra were calibrated to the C 1s peak at 284.5 eV using Avantage 5.9 software. Raman spectroscopy was conducted with a Renishaw Invia Raman microscope.

### 2.4 Working Electrode Preparation

To prepare the electrocatalyst ink, 870 μL of ethylene glycol was mixed with 130 μL of a 1 wt % Nafion solution, followed by the addition of 5 mg of the catalyst powder. The resulting suspension was ultrasonicated for 60 min to ensure uniform dispersion. Then, 3 μL of the ink was drop-cast onto a precleaned glassy carbon electrode (GCE) and dried at 60 °C for 6 h under ambient conditions.

### 2.5 Electrochemical Measurements

Electrochemical performance of the ball-milled catalyst powders was evaluated by using linear sweep voltammetry (LSV) in a three-electrode configuration with a Biologic VSP Potentiostat. A Hg/HgO (1 M KOH) electrode was used as the reference, a graphite rod as the counter electrode, and a 3 mm modified GCE as the working electrode in the 1 M KOH electrolyte. LSV measurements were conducted for both the OER and HER over defined potential ranges, and all potentials were converted to potentials vs reversible hydrogen electrode (RHE), according to the following equation: *E*_RHE_ = *E*_Hg/HgO_ + 0.059 pH + 0.098 V. Stability was assessed using a rotating disk electrode (RDE) via chronopotentiometry at a constant current density of 10 mA cm^–2^. Electrochemical impedance spectroscopy (EIS) was employed to examine charge transfer characteristics, while cyclic voltammetry (CV) was used to calculate the double-layer capacitance (C_dl_) for estimating the electrochemically active surface area (ECSA).

### 2.6 First-Principles Simulations

Spin-polarized density functional theory (SP-DFT) calculations were used to determine the impact that boron had on the HEA crystal structure and the related changes in hydrogen and oxygen adsorption energies. The projector augmented wave method (PAW)^30^ and generalized gradient approximation as the exchange functional (GGA-PBE)^31^ were applied, as implemented in the Vienna ab initio simulation package (VASP)^32^ under the framework of MedeA3.4. The FCC and BCC structures of the HEA were constructed using a special quasi-random structure (SQS) approach, and the boron placement was considered as both interstitial and substitutional. A plane wave cutoff energy of 500 eV was used with a Hellmann–Feynman force convergence of less than 10^–3^ eV/Å, followed by a self-consistency threshold of 10^–6^ eV. A *k*-point spacing of less than 0.25 Å^–1^ was used for all of the models. The boron defect formation energies (Δ*H*) were calculated using eq 1, where *E* ^defect^ and *E* ^pristine^ are the total electronic energy of the cation-bearing structure and pristine cells, respectively. The *n*_*i*_μ_*i*_ term represents the chemical potentials of a defect’s atomic constituents.^33^

1 $${\Delta H}^{defect}=E^{defect}-E^{pristine}-\sum n_{i}\mu_{i}$$

Similarly, to better understand the impact of boron on hydrogen and oxygen adsorption energies, which is an indicator of the HER and OER, the slab approach was used and the adsorption energies on the 100 surfaces of the structures were considered.^34^

## 3 Results and Discussion

### 3.1 Structure, Chemistry, and Morphology of the Synthesized HEA

The XRD patterns presented for Pristine-HEA and B-HEA samples reveal that both compositions exhibit a mixture of FCC and BCC phases, as shown in Figure 1. For the Pristine-HEA sample, the phase composition comprises 77.6% FCC and 22.4% BCC, indicating that the majority of the crystalline phase adopts the FCC structure, while a smaller fraction forms the BCC structure. The crystallite size for the FCC phase is 4.6 nm, suggesting relatively fine grains, whereas the BCC phase exhibits larger crystallites with an average size of 15.2 nm. This disparity suggests that the BCC phase consists of larger or more well-developed grains compared with the FCC phase. The strain within the FCC crystallites is relatively high at 4.6%, indicative of notable lattice distortions. In contrast, the BCC phase displays a lower strain value of 1.6%, reflecting a lower lattice distortion. Regarding the overall material structure, 86.0% of the sample is amorphous, lacking long-range crystalline order, while only 14.0% is crystalline, distributed between the FCC and BCC phases.

**Figure 1:**
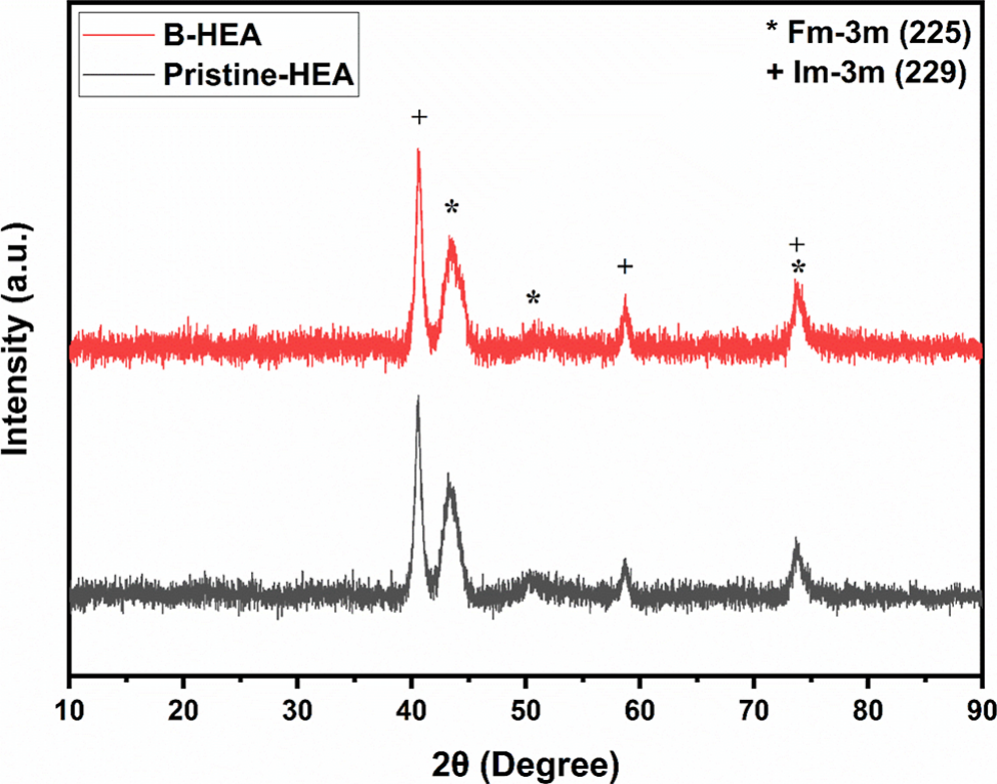
XRD patterns of Pristine-HEA and B-HEA samples.

For the B-HEA sample, the phase composition changes to 73.7% FCC and 26.3% BCC. The addition of boron increases the proportion of the BCC phase compared with the original alloy. The crystallite size for the FCC phase is reduced to 1.7 nm, indicating a much finer microstructure, possibly due to the influence of boron. Similarly, the BCC crystallites are also significantly smaller with a size of 3.6 nm, suggesting a more refined microstructure overall. The strain within the FCC phase slightly increases to 4.9%, indicating higher lattice distortions with the addition of boron. The BCC phase strain shows a higher increase with respect to the original alloy, going up to 3.7%. The amorphous content remains nearly the same at 86.3%, indicating that boron does not significantly affect the overall amorphous nature of the material.^13^ The crystalline content also remains similar at 13.7%, but the ratio of FCC to BCC phases changes with the addition of boron. Related data are listed in Table 1. The Pristine-HEA sample predominantly shows an FCC phase with relatively large BCC crystallites and is mostly amorphous with significant strain in the FCC phase. Adding boron to the alloy increases the proportion of the BCC phase and significantly reduces the crystallite size for both phases.^35,36^ The strain in the FCC phase slightly increases, while the overall amorphous and crystalline contents remain almost unchanged.^36^ These differences suggest that boron plays a crucial role in refining the microstructure, leading to smaller and potentially more stable crystallites while also altering the phase distribution between FCC and BCC structures.^37^ Furthermore, the XRD peaks of the B-HEA sample are shifted to the left side compared to those of the Pristine-HEA sample. This shift indicates that the *d*-spacing of the crystal structure increases with the addition of boron. Boron atoms can occupy interstitial positions in the lattice, which leads to increased internal strain. This increased strain, as measured and detected, results in breaking of the crystal structures, as evidenced by the decrease in crystallite size for both crystal structures. Additionally, the increase in *d*-spacing, shown by the leftward shift of the XRD peaks, reflects an increase in the volume of the crystal structure.^36^

**Table 1:** Structural and Microstructural Parameters of Pristine-HEA and B-HEA Powders Extracted from the XRD Spectrum

	Pristine-HEA	B-HEA
A/C	86/14	86.3/13.7
FCC (%)	77.9	55.5
BCC (%)	22.1	44.5
FCC crystallite size (nm)	4.6	1.7
BCC crystallite size (nm)	15.2	3.6
ε_FCC_ (%)	4.6	4.9
ε_BCC_ (%)	1.6	3.7

This observation is in agreement with the DFT calculations, which demonstrated the extreme likelihood of boron placement in substitutional and interstitial sites of both FCC and BCC structures, with the substitutional B being more likely in both cases.^38–40^ This is evident from the lower enthalpy of formation of the boron-related defects in both structures (Figure 2a). Given that the substitutional B placement in both BCC and FCC demonstrates a lower enthalpy of formation, the thermodynamic drive toward formation of such defects is higher and therefore expected in higher concentration, in comparison to interstitial B. The calculated changes in the lattice parameters due to boron-induced strains show that the lattice should expand upon doping with B in both cases of interstitial and substitutional placement in the FCC crystal. However, we expect contraction of the lattice in the case of the B-substitutional placement in the BCC structure. The addition of substitutional B per formula unit of the HEA in FCC and BCC structures resulted in a +0.2% expansion and −1.6% contraction, respectively. However, for the case of interstitial B, we expect +2.3% and +4.7% volume expansion in FCC and BCC structures, respectively. The experimentally seen higher strain in the FCC structure is also explained through these results. The boron defects in both substitutional and interstitial forms in the FCC structure cause a tensile strain, thus causing a resonant behavior. However, in the BCC case, substitutional B causes compressive and interstitial B introduces the tensile strain, and given the higher expected concentration of the substitutional B, and higher strains of the interstitial B, the same resonant behavior does not form as the strains are in opposing directions. Thus, DFT calculations explain the cause of strain effects in the FCC structure. Moreover, the defect formation energies in all cases are negative, which means from a thermodynamic stability point of view, there exists full solubility for boron atoms in the HEA structure, followed by its phase decomposition due to an unphysical number of B-induced defects. This is clear from the Arrhenius eq 2 of defect concentration *C* with respect to defect formation energy.

2 $$\frac{C}{N}=e^{\left({-\Delta H}^{defect}/kT\right)}$$

Here, *N* refers to the available number of sites for the defect, *k* is the Boltzmann constant, and *T* is the temperature. The $\frac{C}{N}$ ratio less than one indicates limited solubility, unity means complete solubility, and a ratio greater than one indicates not just full solubility but rather an infinite solubility, which is associated with phase decomposition of the structure. Given that the defect formation enthalpies (-Δ*H* ^defect^) shown in Figure 2a are negative, the $\frac{C}{N}$ > 1; boron placement is expected to be rather favorable, with the FCC being more severe than BCC, which is also reflected in the experimentally found dissociation of the FCC structure as well.

**Figure 2:**
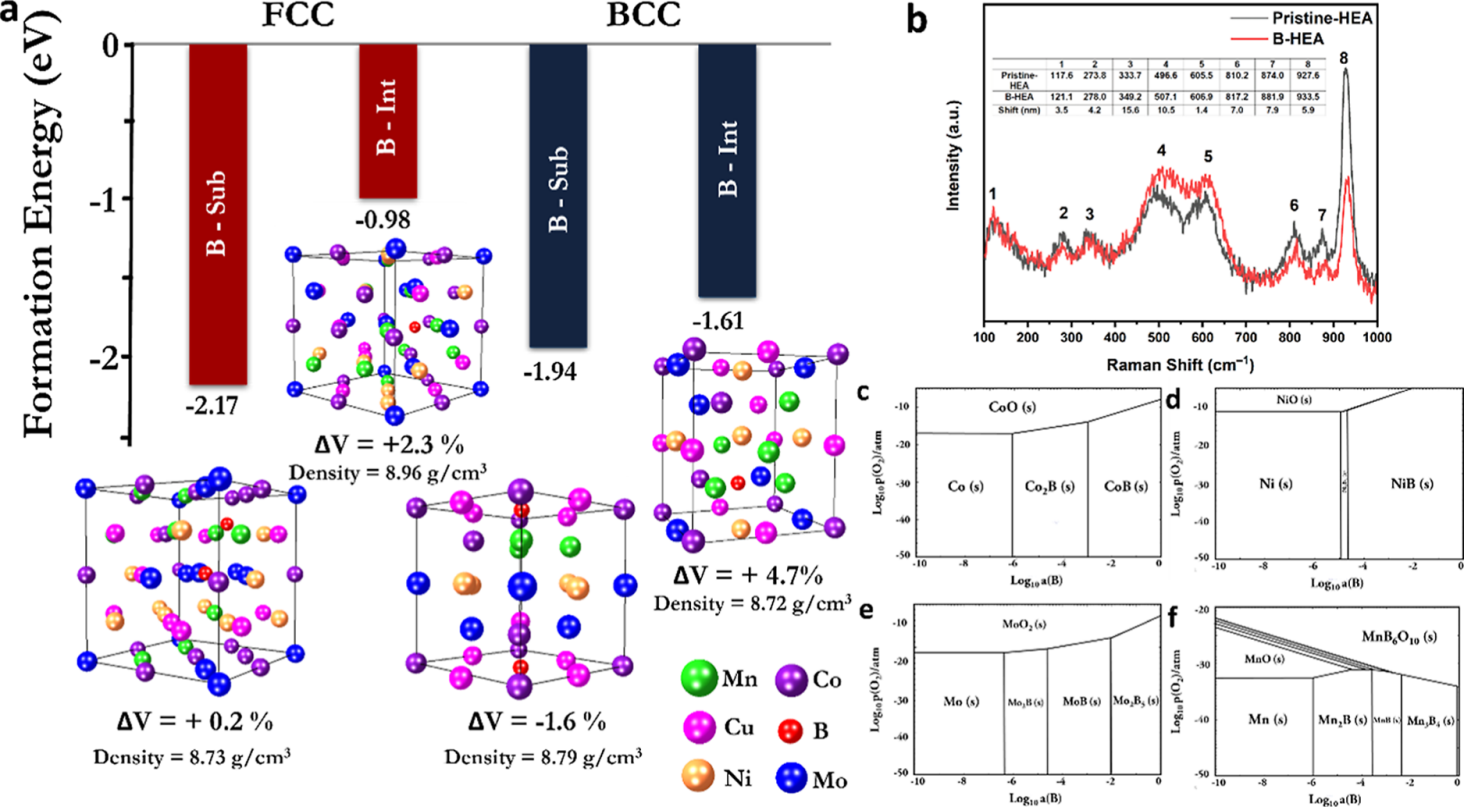
(a) Defect formation enthalpy energies for the substitutional and interstitial B in the FCC and BCC are negative, demonstrating complete solubility, followed by a thermodynamic drive toward phase dissociation as well as the volumetric changes in the lattice due to B-insertion per formula unit of the HEA. (b) The Raman spectrum of Pristine-HEA powders and B-HEA powders demonstrate the same structural features and do not show any formation of borides, which are otherwise expected under the equilibrium thermodynamic conditions as demonstrated by the Kellogg diagrams for the (c) Co–B–O, (d) Ni–B–O, (e) Mo–B–O, and (f) Mn–B–O systems.

The expected phase decompositions are also seen from the Kellogg diagrams achieved through CALPHAD calculations^41^ on the constituents of the HEA, which demonstrate formation of borides upon addition of B. Nevertheless, due to the nonequilibrium nature of the mechanical alloying and the relatively low temperature reaction environment, we only observe B-doping and no phase transformations, preserving a boron-doped HEA structure, as verified by the XRD and Raman analysis of the samples with and without B, which are inherently similar. In other words, the thermodynamic drive for B-insertion and the nature of mechanical alloying give rise to a nonequilibrium structure that is rather unique.

The Raman spectra of Pristine-HEA and B-HEA samples, presented in Figure 2b, were obtained by scanning over 20 distinct points on each sample to ensure reproducibility and account for sample heterogeneity. The spectra revealed two distinct patterns. The first type exhibited a featureless profile typical of metallic systems with low Raman activity, confirming the metallic nature of the HEAs. The second type displayed sharper peaks at specific wavenumbers: 106.23, 273.12, 348.30, 498.37, 623.19, 813.76, 875.14, and 932.32 cm^–1^ for Pristine-HEA and 106.25, 274.07, 349.32, 514.30, 639.18, 814.05, 880.32, and 939.13 cm^–1^ for B-HEA. These peaks are attributed to the presence of some oxide phases formed during the high-energy ball milling.

Despite the similarity in the peak sets between the two compositions, B-HEA exhibits a systematic shift of these peaks toward higher wavenumbers compared to the undoped sample. This shift suggests an increase in the internal lattice strain caused by the incorporation of boron into the HEA matrix. This observation aligns with the findings from XRD analysis,^42^ further supporting the hypothesis that boron doping introduces local distortions into the crystal lattice. Moreover, the absence of Raman peaks corresponding to boride phases reinforces the conclusion that boron does not form separate boride compounds but instead is doped into the HEA solid solution. This is consistent with the expected behavior of boron, as a minor alloying element in the HEA system.

The FE-SEM images of the mechanically alloyed powders are shown in Figure 3. The morphology of the mechanically processed HEA powders reveals that both compositions of powders exhibit a similar planar structure. Upon closer examination of these planes, it becomes evident that each consists of a combination of lamellar (layered) structures and smaller particles. This means that the surface or the cross sections of these powders have flat or sheet-like arrangements. Within these planes, there are both layered formations and smaller individual particles. This consistent structural pattern indicates that the mechanical alloying methods used result in a distinctive and uniform morphology across Pristine-HEA and B-HEA samples. Furthermore, the general appearance indicates that the particle size of the B-HEA powders is smaller than that of the Pristine-HEA sample. This observation aligns with the XRD quantitative results, which show that the crystallite size decreases with the addition of boron.

**Figure 3:**
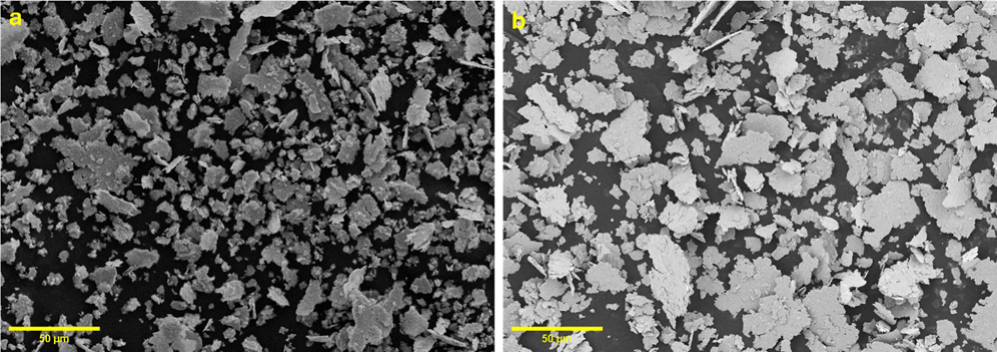
FE-SEM images of the (a) NiCoCuMoMn and (b) B-doped NiCoCuMoMn HEA powders.

The TEM elemental mappings of both Pristine-HEA and B-HEA samples, which are shown in Figure 4, indicate that all elements are uniformly distributed throughout the powders. Boron is not detectable by TEM mapping due to its low atomic weight and is therefore not shown here.

**Figure 4:**
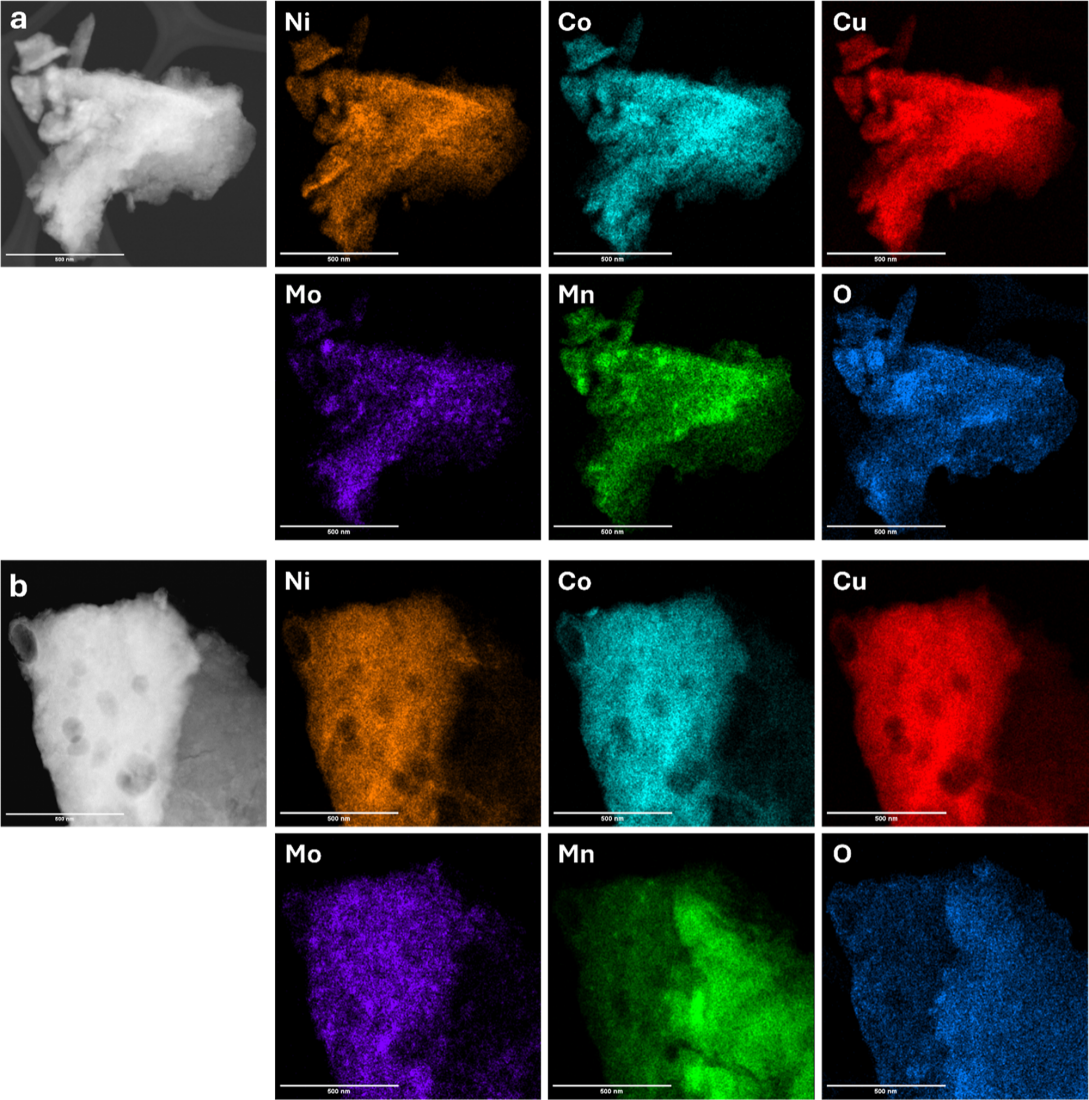
HRTEM images and elemental mapping of (a) Pristine-HEA and (b) B-HEA samples.

The SAED patterns of Pristine-HEA and B-HEA powders as shown in Figure 5 revealed their respective *d*-spacing values. For the Pristine-HEA powders, *d*-spacings of 2.1 and 1.9 Å were determined for the *Fm*3̅*m* space group, corresponding to the (111) and (200) planes, respectively. Additionally, *d*-spacings of 1.6 Å and 1.3 Å were identified for the *Im*3̅*m* space group, associated with the (200) and (211) planes. In the case of B-HEA powders, the *d*-spacings exhibited slight variations. The values were calculated as 2.1 Å and 1.8 Å for the *Fm*3̅*m* space group with the (111) and (200) planes, while *d*-spacings of 1.5 Å and 1.3 Å were observed for the *Im*3̅*m* space group with the (200) and (211) planes, respectively. These measurements show strong agreement with the *d*-spacings extracted from XRD spectra, confirming the consistency and reliability of the structural data. Boron doping was found to induce a noticeable alteration in the lattice parameters of both FCC and BCC crystal structures, as evidenced by the small shifts in *d*-spacing values. This lattice expansion suggests that boron introduces localized distortions and internal strain within the HEA matrix, aligning with prior observations from XRD analyses and reinforcing the role of boron in modifying the crystal structure. The *d*-spacing calculation from HRTEM by the TTF method shows that the *d*-spacing for Pristine-HEA is 2.3 Å, while the *d*-spacing for B-HEA is 2.3 Å, which is consistent with the SAED and XRD spectrum results. The addition of boron causes the lattice crystal to expand.

**Figure 5:**
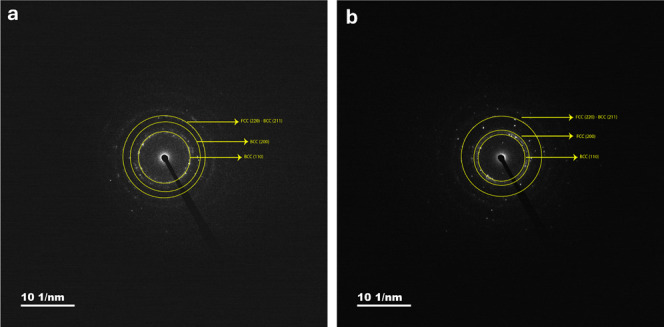
SAED patterns of (a) Pristine-HEA powders and (b) B-HEA powders.

To further study the surface composition of the powder samples, the high-resolution XPS spectra of all the elements present in the undoped and doped samples are provided in Figures S3 and 8, respectively. As shown in Table S1, XRF analysis confirmed a homogeneous distribution of the principal metallic elements (Ni, Co, Cu, Mn, and Mo) in both pristine and B-doped HEA powders, with near-equiatomic ratios (∼20 at. %), indicating uniform alloy formation. However, because XRF and EDS are not sensitive to light elements, such as boron, complementary ICP–MS and XPS analyses were employed for accurate boron quantification. ICP–MS revealed a composition of Mn (18.2 at. %), Co (18.4 at. %), Ni (18.1 at. %), Cu (17.6 at. %), and Mo (18.0 at. %), along with 1.74 wt % boron, confirming successful bulk incorporation of B into the alloy matrix. Surface-sensitive XPS further verified the presence of boron, showing 2.4 at. %, which is lower than the bulk value due to partial oxidation and limited surface segregation. These complementary analyses collectively confirm that boron is effectively doped throughout the HEA structure, influencing both the surface and bulk chemical environments.

**Figure 6:**
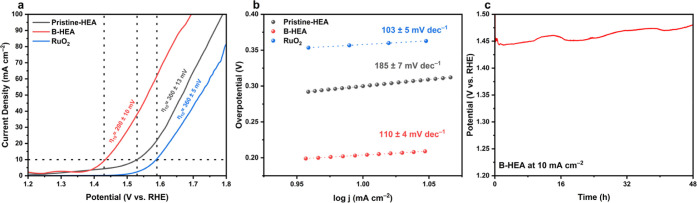
(a) LSV curves related to the OER of Pristine-HEA, B-HEA, and RuO_2_ electrocatalysts, (b) the corresponding Tafel slopes for the OER, and (c) the chronopotentiometry curve demonstrating the long-term (48 h) stability of the B-HEA sample at a current density of 10 mA cm^–2^.

**Figure 7:**
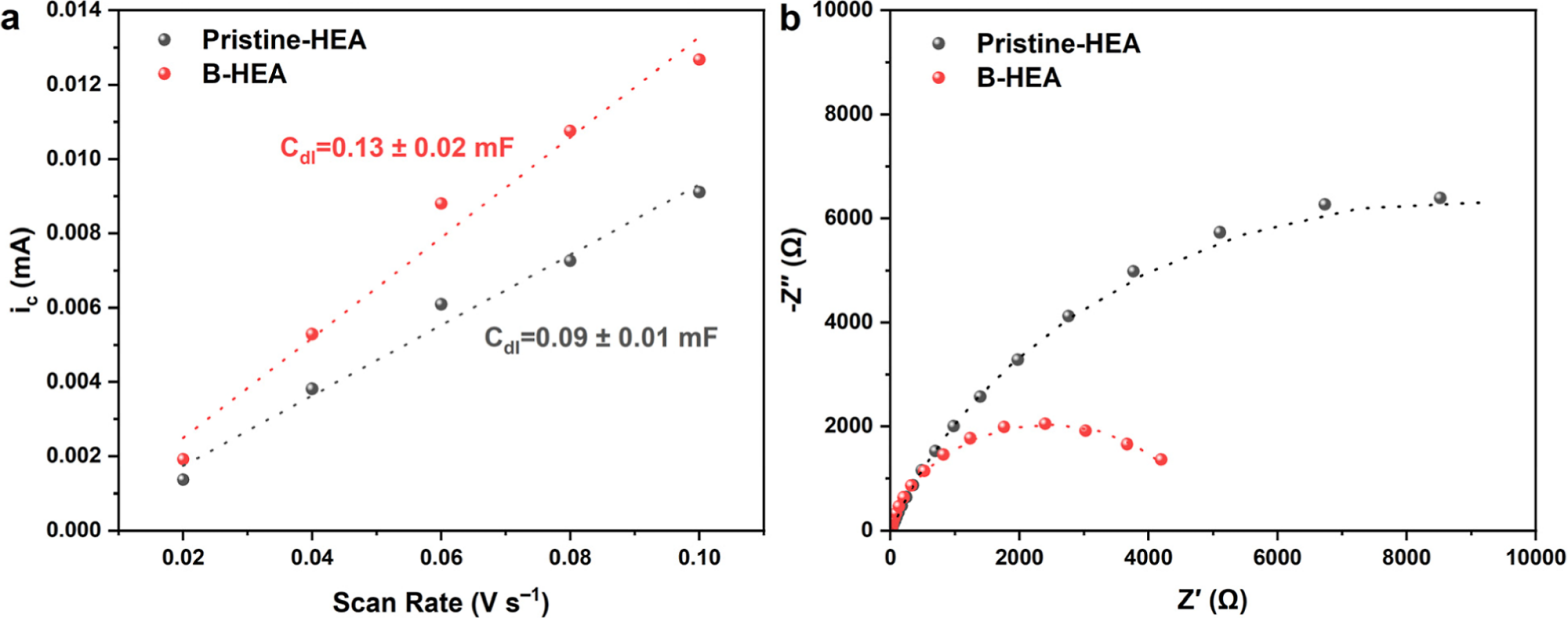
(a) Nonfaradaic current density versus scan rate curves for Pristine-HEA and B-HEA samples, linearly fitted for ECSA calculations, and (b) EIS Nyquist plots for Pristine-HEA and B-HEA-modified electrodes.

**Figure 8:**
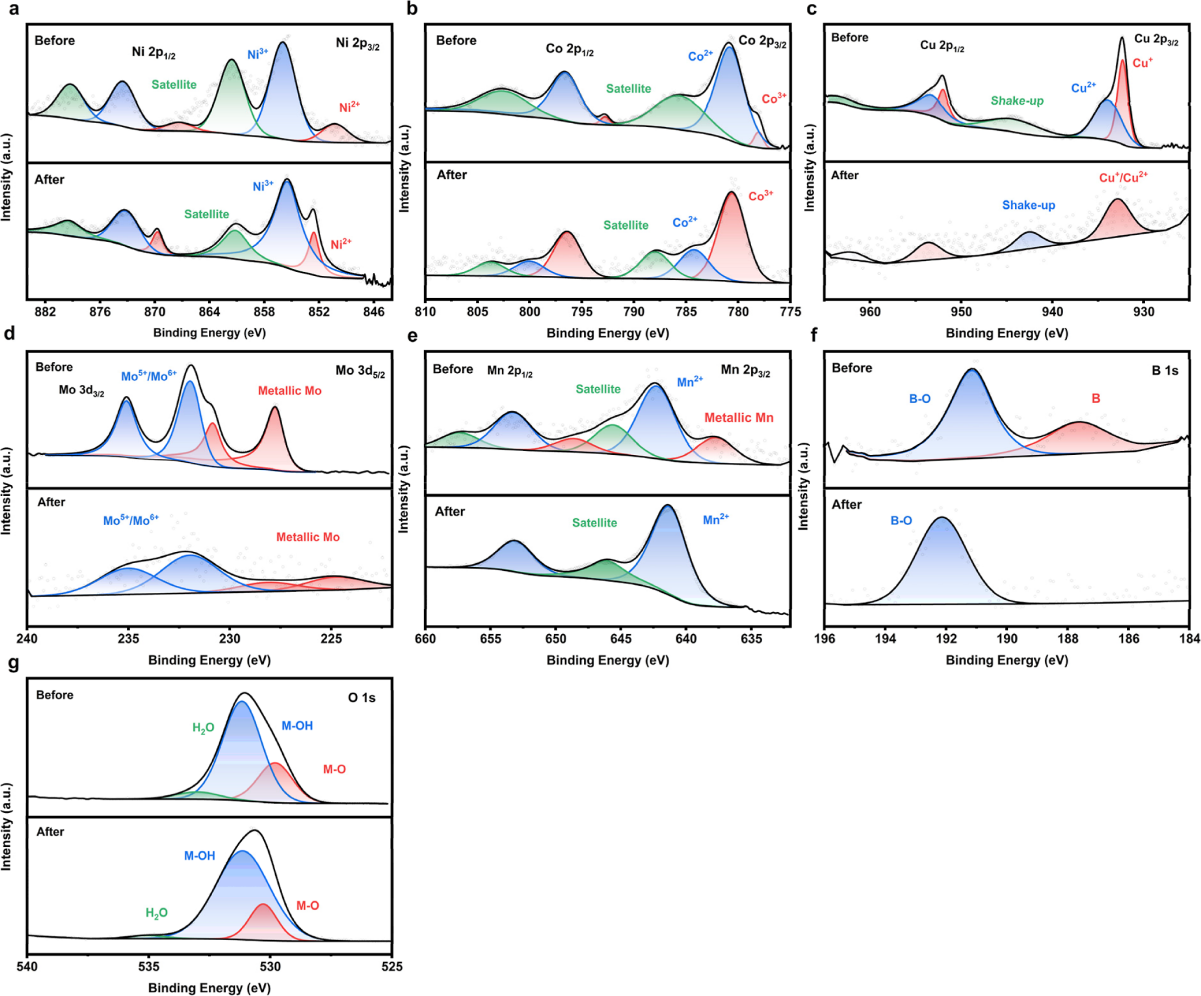
High-resolution XPS spectra of (a) Ni 2p, (b) Co 2p, (c) Cu 2p, (d) Mo 3d, (e) Mn 2p, (f) B 1s, and (g) O 1s for the B-HEA sample before and after long-term OER measurements.

### 3.2 Electrochemical Measurements

By carefully selecting the elements, controlling their proportions, and optimizing doping strategies, HEAs show great potential in the development of sustainable energy technologies, particularly in catalysis for water splitting—a process involving the OER and HER that is crucial for hydrogen production. By minimizing energy losses and lowering the overpotentials required for these reactions, HEAs can serve as efficient catalysts, thereby improving the overall energy conversion efficiency and contributing to the economic feasibility of hydrogen production as a clean energy source. Here, we have explored the activity of Pristine and B-HEA samples toward the electrocatalytic OER and HER.

#### 3.2.1 OER Activity

The OER activity of the HEA before and after boron doping was evaluated in a 1 M KOH electrolyte saturated with O_2_. As seen in Figure 6a, LSV plots show the current density against the electrode potential vs RHE, highlighting the OER onset overpotential of the electrodes before and after boron doping. To gain further information about the OER kinetics, the slopes of the Tafel plots and their corresponding Tafel slopes are considered, as shown in Figure 6b. The Tafel slopes can be obtained from the LSV curve by the following equation^43^

3 η=a+blog⁡j

where η, j, and b represent the overpotential, current density, and Tafel slope, respectively.

The overpotential (η_10_) to achieve a current density of 10 mA cm^–2^ for the Pristine-HEA and B-HEA samples is 300 ± 13 mV and 200 ± 10 mV, respectively, both surpassing the activity of RuO_2_ with a η_10_ of 360 ± 5 mV. Pristine-HEA and B-HEA samples exhibit a Tafel slope of 185 ± 7 and 110 ± 4 mV dec^–1^, respectively, which displays an enhancement in the kinetics of the OER after the addition of boron atoms. Regarding the mechanisms of OER enhancement, the presence of boron atoms promotes the transition of active metal sites to higher oxidation states at lower working potentials, therefore providing active sites for the OER and improving the overpotential.^29^ OER performance is improved as the high electronegativity of boron is combined with transition metals, leading to weakening of metal–metal bonds and hence enhancing charge transfer and oxygen intermediate adsorption.^44^ Specifically, it has been shown that the coupling of Co and B atoms can lead to the adjustment of the cobalt oxide structure and thereby improve OER activity.^44^ More detailed discussions around the mechanisms of the OER are included in the computational and postcharacterization studies.

Furthermore, the long-term stability measurements were carried out at a constant current density of 10 mA cm^–2^ for the B-HEA sample (Figure 6c), where the electrode showed a stable overpotential for 48 h. These experiments reveal that the addition of boron atoms has led to a decrease in both overpotential and Tafel slope while maintaining extraordinary stability.

#### 3.2.2 HER Activity

Similar to what was observed in OER measurements, the electrocatalysts were prepared for HER analysis on GC electrodes, and a negative potential was applied in 1 M KOH electrolyte. The corresponding LSV graphs and their Tafel plots are displayed in Figure S1. Pristine-HEA and B-HEA samples show overpotentials of 525 ± 24 and 593 ± 44 mV, respectively. Unlike OER measurements, the overpotential required to achieve 10 mA cm^–2^ increases after the addition of boron atoms to the structure. To study the HER kinetics and determine the main mechanism, the Tafel slopes are calculated as 185 ± 1 and 206 ± 3 mV dec^–1^ for HEA and B-HEA, respectively, showing a decrease in the HER kinetics. The boron atom exhibits moderate electron-withdrawing characteristics, which generally lowers the metal d-band center and consequently weakens hydrogen adsorption (increasing ΔG_H*_), thereby elevating the HER overpotential.^45,46^ This trend is also confirmed by the DFT results, discussed in detail in the following sections. Similar HER-suppressing effects of boron have been reported in other catalytic processes, such as the nitrogen reduction reaction (NRR).^47^

#### 3.2.3 ECSA and EIS Measurements

The ECSA of samples was approximated with the double-layer capacitance (C_dl_) method. To do so, CV measurements were done in the nonfaradaic potential region at five different scan rates in the range of 20–100 mV s^–1^. The corresponding CV curves are displayed in Figure S2. ECSA values were subsequently calculated according to the following equation

4 $$ECSA=C_{dl}/C_{s}$$

where *C*_s_ represents the specific capacitance of the material and *C*_dl_ is the double-layer capacitance estimated from the *i*_*c*_ vs ν plot according to the following equation

5 $$i_{c}=\nu C_{dl}$$

where *i*_*c*_ is the charging current and ν is the scan rate.

It has been shown that the average value of *C*_s_ in 1 M KOH for metallic surface can be assumed as 0.040 mF cm^–2^.^48,49^ According to Figures 7 and 8, the double-layer capacitance values for the samples with and without boron are calculated as 0.09 ± 0.01 and 0.13 ± 0.02 mF, respectively. It is seen that the sample with boron doping shows a higher C_dl_ and thus a higher ECSA of 3.25 ± 0.50 cm^2^ compared to the undoped sample with an ECSA of 2.25 ± 0.25 cm^2^, which means a higher abundance of electrochemically active sites promoting the OER. Finally, the EIS technique was employed to explore the interfacial behavior of the HEA with and without boron doping. In this case, the high-frequency resistance indicates the solution resistance (*R*_s_), while the arc radius of the Nyquist plot represents the charge transfer resistance (*R*_ct_).^43,50,51^ As expected from the previous electrochemical measurements, the boron-doped sample shows a lower *R*_ct_ of 4861 ± 23 Ω compared to that of the sample without boron, around 17654 ± 53 Ω. The detailed results of EIS measurements and the corresponding fit results are listed in Table 2.

**Table 2:** EIS Fitting Data for All Specimens Obtained Using ZView Software

sample	R_s_ (Ω)	R_ct_ (Ω)	CPE-T (μΩ ^–1^ s^n^)s	CPE-P
Pristine-HEA	12.9 ± 0.3	17654 ± 53	7.7 × 10^–5^	0.8
B-HEA	12.3 ± 0.7	4861 ± 23	10^–4^	0.9

#### 3.2.4 Poststability Characterization

The high-resolution XPS analysis of the B-HEA sample before and after long-term stability testing (e.g., 48 h of chronopotentiometry) reveals significant insights into surface chemical states and structural reconstruction under the OER conditions. In the Ni 2p spectrum, the Ni^2+^ peak shifts from 850.48 to 852.78 eV after stability measurements, indicating partial surface oxidation and electron redistribution, potentially due to Ni leaching and mild lattice reconstruction. Notably, the Ni^3+^ peak remains relatively constant around 855 eV, suggesting that while oxidation progresses, the higher oxidation state is stabilized during the OER.

In the Co 2p region, the Co^3+^ peak shifts significantly from 777.98 to 780.78 eV, and the Co^2+^ peak shifts from 781.18 to 784.18 eV. This marked shift is accompanied by an intensity increase of Co^3+^ and a decrease in Co^2+^, clearly indicating a progressive oxidation of cobalt species. Such a transformation implies a surface reconstruction in which Co^2+^ is oxidized to the more OER-active Co^3+^, consistent with established mechanisms involving the dynamic evolution of transition metal sites under electrochemical stress.

For Cu 2p, Cu^+^ and Cu^2+^ peaks are detected at 932.31 and 934.13 eV, respectively, before the stability test. Poststability, the Cu signals weaken and do not exhibit noticeable shifts, suggesting partial dissolution of copper species into the electrolyte, a known phenomenon for Cu-based systems under anodic polarization, which limits its long-term contribution to catalytic activity.

Mo 3d spectra reveal the presence of metallic Mo and mixed-valence oxides (Mo^5+^/Mo^6+^) before the test. After the long-term operation, these peaks persist but with significantly reduced intensity, implying the loss of Mo from the surface, likely through oxidative dissolution—a common degradation route for Mo-based components under alkaline OER conditions.

Mn 2p spectra initially show both metallic Mn (637.76 eV) and Mn^2+^ (642.30 eV). However, after 48 h of operation, only a strong Mn^2+^ peak is observed, highlighting extensive surface oxidation and possibly the formation of a passivating Mn­(OH)_2_ or MnO layer. This transformation aligns with reports that Mn can undergo rapid surface redox reactions, leading to higher valence states that stabilize as Mn^2+^ in alkaline media.

The B 1s spectrum before the OER shows peaks at 187.57 and 191.15 eV, corresponding to elemental B and B_2_O_3_, respectively. The quantitative analysis of the B 1s spectrum shows the presence of 2.4 at. % boron in the structure. After electrochemical treatment, the elemental B peak disappears, and a shifted B–O peak emerges at 192.1 eV, indicating complete surface oxidation of boron. This shift to higher binding energy reflects stronger B–O bonding environments, possibly forming BO_*x*_ clusters under anodic bias.

Finally, the O 1s spectra show a slight shift to lower binding energies poststability, transitioning from surface hydroxyl (M–OH) to metal–oxygen (M–O) bonds. This evolution suggests deprotonation of hydroxyl groups and the formation of more robust oxide/hydroxide layers, which are commonly associated with enhanced catalytic durability. The oxygen content on the Pristine-HEA powders and B-HEA powder samples indicates the presence of oxide surfaces, with measured amounts of 41.1% and 42.1%, respectively. It could be deduced that the addition of boron to the HEA structure has increased the surface oxidation of the sample after mechanical alloying.

#### 3.2.5 Computational Studies

To complement the experimental observations and provide a mechanistic understanding of the electrocatalytic performance, DFT calculations were carried out. The enhancement in the OER overpotential is also predicted by the DFT-calculated results, which show a reduction in the oxygen adsorption energy upon addition of boron. It is displayed that the boron atoms do not position themselves in the plane of other metals but rather create an angular alignment in an otherwise isoplanar site (Figure 9a). This phenomenon creates a potential well on the surface, which tends to become an adsorbate trap site, acting as a quasi-vacancy (see Figure 9b), irrespective of the chemical thermodynamic reservoirs, which are evident from Figure 9c,d, where the chemical potentials of the O and H are kept as variables. The calculation of the most probable site for B had made it clear that boron is energetically favored in substitutional sites (Figure 2a). Based on the structure relaxation of the B-substitutional supercells, we find that boron deviates from the plane of metals, creating metal–boron–metal angles of 96°, 141°, and 165°, which results in formation of an open space that very much resembles a vacancy. This quasi-vacancy space, in turn, acts as a trap site for the potential adsorbates, as shown here. The deviation also creates distortion, which is clear from the pair correlation functions retrieved from the structures before and after relaxation. This phenomenon of boron-induced quasi-vacancies is rather unique and highly desirable when it comes to making adsorptions more likely and enhancing the surface reactivity. It is noteworthy that the adsorption of atomic oxygen, which was calculated through DFT, is a direct indicator of OH adsorption on the surface, given that OH binds through the partial electron density of oxygen. The same is true for the O* that are intermediate reaction steps of the OER,^52^ thus giving an indirect indication of the OER overpotential.

**Figure 9:**
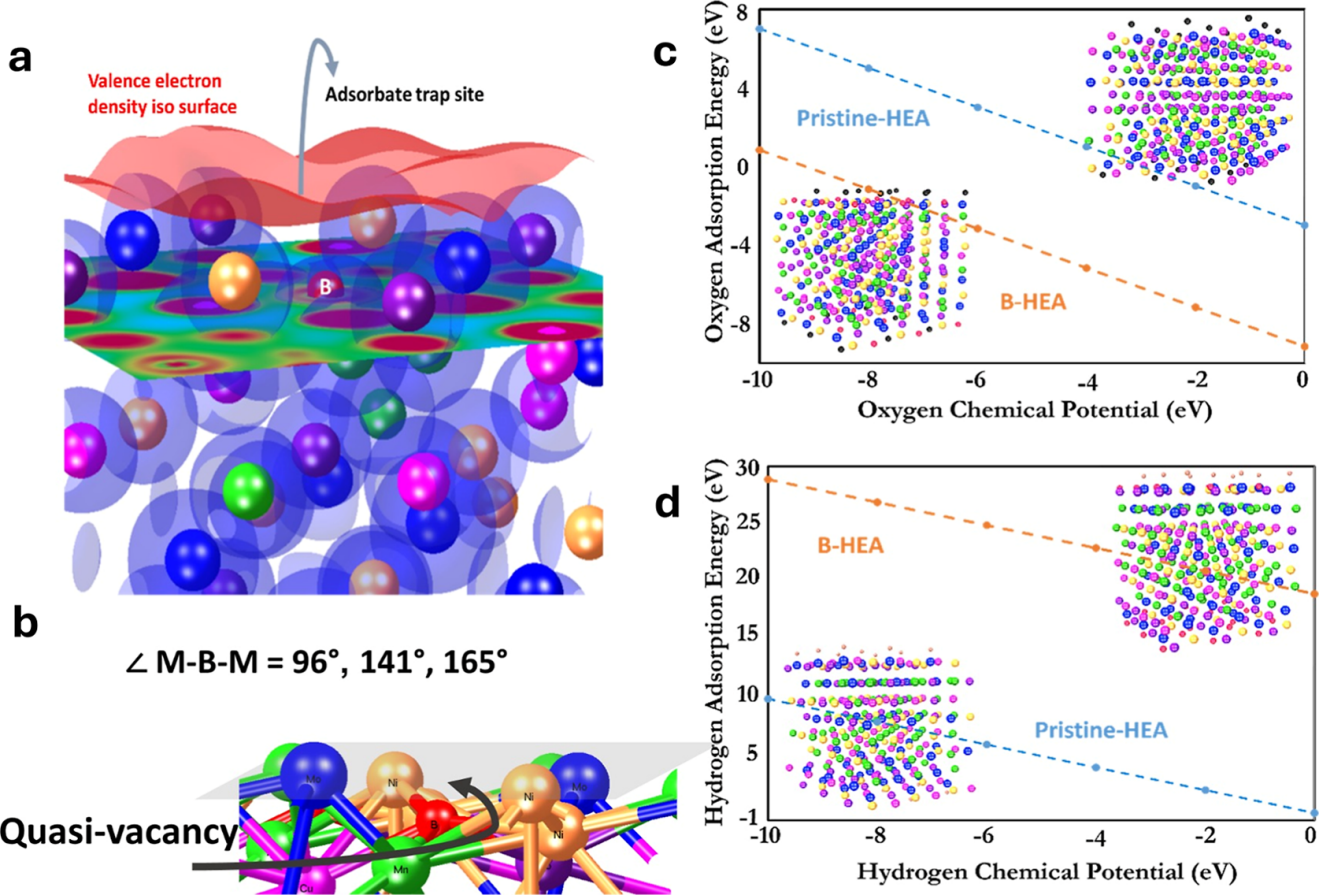
(a) Schematic of adsorption sites available after the introduction of boron, (b) the deviations of the isoplanar metals due to the presence of B that results in a quasi-vacancy, and adsorption energies predicted by DFT calculations for (c) oxygen and (d) hydrogen.

This increase in the HER overpotential and the respective Tafel slope is also explained by the DFT results, which show a very dramatic increase in the H-adsorption energy upon addition of boron (Figure 9d). Moreover, the DFT results of the H-adsorption are positive, with and without boron, which means H-adsorption is endothermic and not spontaneously likely. Therefore, the HER process happens in the first place due to the defective nature of the structure, which must be localized, where H-adsorption energies are not positive (such as vacancy sites). However, in the absence of any surface imperfections, we calculated an increased H-adsorption energy upon boron doping, which explains the increased HER overpotentials. Based on the calculated DFT results, we predict that the Volmer step is the limiting factor and the appearance of any HER is attributed to the nature of mechanical alloying that leaves behind reactive surfaces due to localized point defects.

## 4 Conclusion

This study demonstrates that boron doping effectively engineers the structure and activity of the NiCoCuMoMn HEAs for water splitting. B incorporation increases the BCC phase fraction (22.1% → 44.5%), refines the FCC crystallite size (4.6 → 1.7 nm), and introduces lattice distortions, as confirmed by XRD, Raman, and TEM. DFT calculations reveal that boron preferentially occupies substitutional sites, creating quasi-vacancy-like centers that enhance oxygen adsorption and charge transfer. Electrochemical analysis shows a substantial OER improvement, with the overpotential reduced from 300 ± 13 to 200 ± 10 mV, Tafel slope from 185 ± 7 to 110 ± 4 mV dec^–1^, and charge transfer resistance from 17654 ± 53 to 4861 ± 23 Ω, alongside an ECSA increase from 2.25 ± 0.25 to 3.25 ± 0.50 cm^2^. The B-doped HEA also exhibits excellent stability over 48 h. In contrast, the HER activity slightly decreases, consistent with DFT predictions of higher hydrogen adsorption energy. In addition to its high catalytic efficiency, the B-doped HEA demonstrates remarkable long-term operational stability, maintaining the OER performance over 48 h without noticeable degradation, confirming its structural robustness under alkaline conditions. The synthesis approach, based on scalable alloy preparation and a simple doping treatment, offers a feasible pathway for large-scale catalyst production. Furthermore, the use of earth-abundant transition metals and boron, a low-cost dopant, underscores the economic viability of this strategy for practical water-splitting applications. Overall, this work establishes boron doping as a tunable design strategy to modulate the phase composition, lattice strain, and adsorption energetics in HEAs. Future efforts will focus on optimizing boron content, exploring codoping, and conducting in situ studies to deepen the mechanistic understanding and extend this approach to other HEA systems for sustainable hydrogen generation.

## Supplementary Material



## Notes

XRF chemical composition data of Pristine-HEA and B-HEA; HER polarization and Tafel plots; ECSA cyclic voltammograms; and XPS spectra of Pristine-HEA (PDF)
