# Supplementary material for: Boron-Doped NiCoCuMoMn High-Entropy Alloys for Enhanced Electrocatalytic Water Splitting: An Experimental and Computational Study
Source: ACS Appl Energy Mater. 2025 Dec 3;8(24):17793–804. doi: 10.1021/acsaem.5c02722 (PMC12728800; doi:10.1021/acsaem.5c02722)
Supplement: Supplementary file 1 [file ae5c02722_si_001.pdf]

Supporting Information

Boron-doped NiCoCuMoMn High-entropy Alloys for Enhanced Electrocatalytic Water Splitting: An Experimental and Computational Study

Hossein Mahdavi[a], *Maryam Mansoor*[b], *Onur Ergen*[c], *Uğur Ünal**[d,e,f], *Hadi Jahangiri*[d*]

[a] Materials Science and Engineering, Koç University, Sariyer, 34450, Istanbul, Türkiye

[b] Energy Institute, Istanbul Technical University, Maslak, 34469, Istanbul, Türkiye

[c] Department of Electronics and Communications Engineering, Istanbul Technical University, Maslak, 34469, Istanbul, Türkiye

[d] Koç University Surface Science and Technology Center (KUYTAM), Koç University, Sariyer, 34450, Istanbul, Türkiye

[e] Department of Chemistry, Koç University, Sariyer, 34450, Istanbul, Türkiye

[f] Koç University Hydrogen Technologies Center (KUHyTech), Koç University, Sariyer, 34450, Istanbul, Türkiye

Corresponding Author: Hadi Jahangiri

hjahangiri@ku.edu.tr

Corresponding Author: Uğur Ünal

ugunal@ku.edu.tr

Table S1. Chemical composition of the synthesized NiCoCuMoMn and B-doped NiCoCuMoMn HEA powders measured by XRF.

	Atomic Percent (%)	
**Element**	**NiCoCuMoMn HEA powder**	**B-doped NiCoCuMoMn HEA powder**
**Ni**	19.95	19.87
**Co**	19.43	19.63
**Cu**	19.96	20.13
**Mo**	20.31	20.11
**Mn**	20.35	20.26

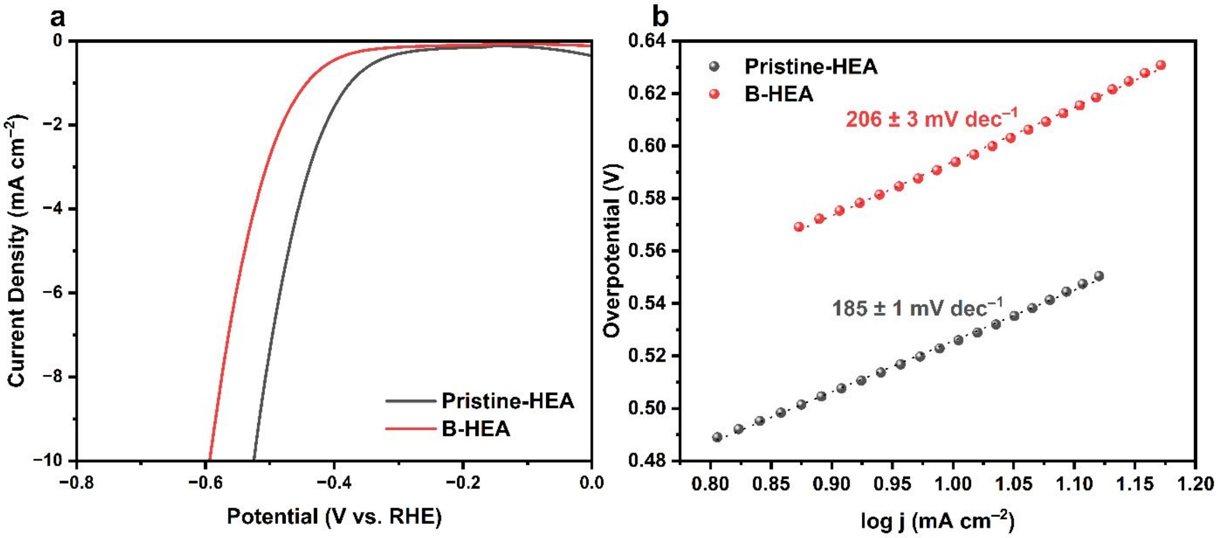

Figure S1 LSV curves related to the HER of Pristine-HEA and B-HEA samples, b) the corresponding Tafel slopes for HER.

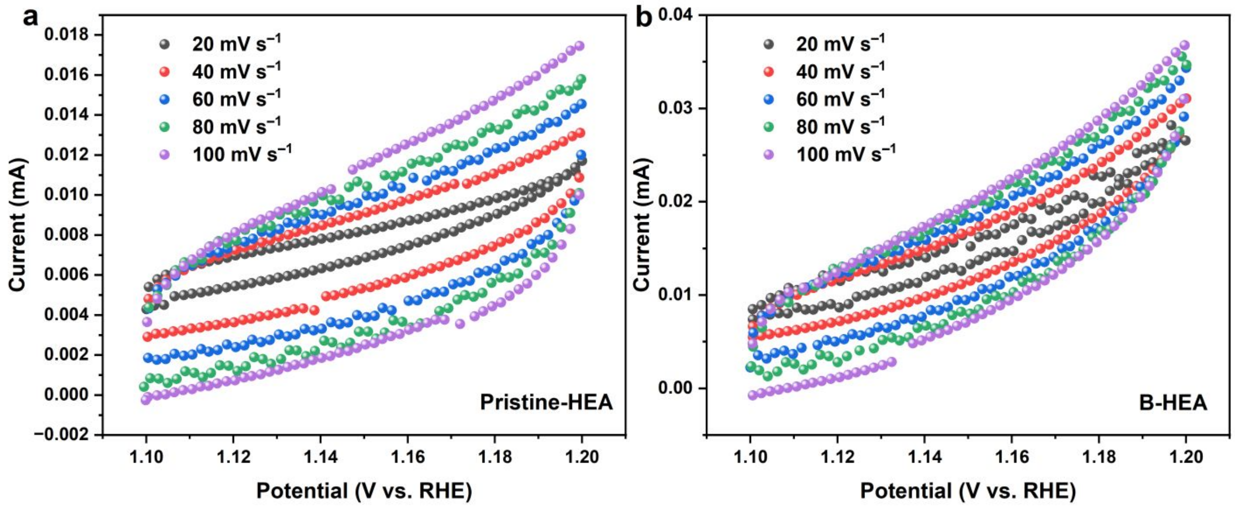

Figure S2 CV curves at between 1.1 and 1.2 V vs. RHE at scan rates ranging from 20 to 100 mV s^{-1} for ECSA.

To explore the surface oxidation state and chemical composition of the HEA sample X-ray Photoelectron Spectroscopy (XPS) was employed and high resolutions spectra corresponding to Ni 2p, Co 2p, Cu 2p, Mo 2p, and Mn 2p are presented are displayed in Figure S3. In the high-resolution spectrum of Ni 2p, the peak observed at 852.7 eV belongs to metallic Ni. Additionally, a NiO peak was detected at 855.7 eV, indicating that both metallic and oxide phases of Ni were present on the surface of the sample, suggesting partial surface oxidation occurred during ball milling. The Co 2p spectrum shows three separate doublets corresponding to metallic Co, Co^{2+}, and satellite peaks. The low-intensity peak observed at 778.2 eV is attributed to metallic Co, while the peak emerging at 781 eV is due to higher oxidation states of Co, namely CoO and Co_2O_3. The presence of the Co^{2+} satellite peak is associated with monopole charge-transfer transitions. In the high-resolution spectrum of Cu 2p, both metallic Cu and Cu^{2+} peaks are detected after deconvolution. The binding energies for metallic Cu were measured at 932.4 eV and 952 eV, while the CuO peaks appeared at 934.8 eV and 954.2 eV. In the Mn 2p spectra, both metallic and oxide peaks are observed. The peak at 640.4 eV is attributed to Mn^0. Additionally, three different oxide peaks are observed in the spectrum corresponding to Mn^{2+}, Mn^{3+}, and Mn^{4+}. The Mo 3d spectrum shows peaks corresponding to metallic Mo and Mo^{5+}. The metallic Mo peak appears as a doublet at 227.7 and 230.7 eV, while the oxide peak corresponding to Mo^{5+} appears at 231.9 and 235 eV.

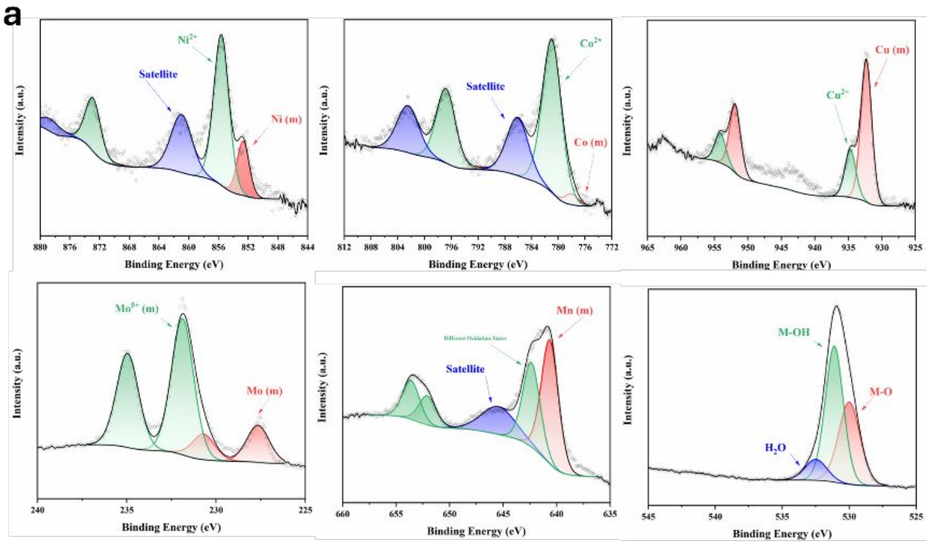

Figure S3 High-resolution XPS spectra of Pristine-HEA sample.
